# Influence of Distal Resistance and Proximal Stiffness on Hemodynamics and RV Afterload in Progression and Treatments of Pulmonary Hypertension: A Computational Study with Validation Using Animal Models

**DOI:** 10.1155/2013/618326

**Published:** 2013-11-10

**Authors:** Zhenbi Su, Wei Tan, Robin Shandas, Kendall S. Hunter

**Affiliations:** ^1^Department of Mechanical Engineering, University of Colorado at Boulder, Boulder, CO 80309, USA; ^2^Department of Bioengineering, University of Colorado Denver Anschutz Medical Campus, Aurora, CO 80045, USA

## Abstract

We develop a simple computational model based on measurements from a hypoxic neonatal calf model of pulmonary hypertension (PH) to investigate the interplay between vascular and ventricular measures in the setting of progressive PH. Model parameters were obtained directly from *in vivo* and *ex vivo* measurements of neonatal calves. Seventeen sets of model-predicted impedance and mean pulmonary arterial pressure (mPAP) show good agreement with the animal measurements, thereby validating the model. Next, we considered a predictive model in which three parameters, PVR, elastic modulus (EM), and arterial thickness, were varied singly from one simulation to the next to study their individual roles in PH progression. Finally, we used the model to predict the individual impacts of clinical (vasodilatory) and theoretical (compliance increasing) PH treatments on improving pulmonary hemodynamics. Our model (1) displayed excellent patient-specific agreement with measured global pulmonary parameters; (2) quantified relationships between PVR and mean pressure and PVS and pulse pressure, as well as studiying the right ventricular (RV) afterload, which could be measured as a hydraulic load calculated from spectral analysis of pulmonary artery pressure and flow waves; (3) qualitatively confirmed the derangement of vascular wall shear stress in progressive PH; and (4) established that decreasing proximal vascular stiffness through a theoretical treatment of reversing proximal vascular remodeling could decrease RV afterload.

## 1. Introduction

Pulmonary hypertension (PH) is associated with a progressive increase of pulmonary vascular resistance (PVR) and sustained elevation of mean pulmonary artery pressure (mPAP), which together contribute to the right heart dysfunction. However, recent studies showed that mPAP apparently does not correlate with either the severity of symptoms or survival. Although many recent developments employing clinical and experimental measurements have investigated symptoms [1–4] to further elucidate the roles of pulmonary arterial mechanics and hemodynamics, the individual relative importance of the components of PVR, pulmonary vascular stiffness (PVS), geometry and cardiac function are unclear in disease progression. In addition, while current PH treatment focuses on distal vasodilation, it is unknown whether or not other treatment targets might beneficially impact right ventricular function, and as a result improve of PH treatment.

Clinical and traditional scientific studies of PH focus on changes in the distal circulation. For example, the distal pulmonary vascular bed can undergo remodeling resulting from growth of smooth-muscle cells, which narrow and restrict distal pulmonary vascular geometry. Additionally lumen obliteration, medial hypertrophy, and adventitial proliferation contribute to the distal diameter decrease and thickness increase in PH [5–7]. In severe PH, a layer of myofibroblasts and extracellular matrix also develops between the endothelium and internal elastic lamina, further thickening and narrowing distal pulmonary arterial walls [8]. The effects of these changes, along with persistent vasodilation, are most clearly seen in decreasing right ventricular (RV) cardiac output and increasing mPAP [9–11], which together indicate increased PVR. In turn, PVR is believed to well quantify RV afterload. These pathologies are currently treated clinically with vasodilators, and further identify the traditional association between distal geometry, PVR and RV afterload, and RV failure in PH progression.

Although vascular distal remodeling and distal vessel vasculopathy are generally considered the only major contributors to PH progression [12–15], recent clinical [2, 16, 17] and basic science [1, 18, 19] research have respectively found that proximal vascular stiffness is more predictive of disease outcomes than PVR and that significant remodeling occurs in the proximal vessels of animal models of the disease. Remodeling chronically alters the stiffness of the proximal arteries through combined changes in their extracellular matrix composition and thickness [1]. Proximal stiffness has been shown to (1) regulate pressure and flow wave velocities in the pulmonary bed [20]; (2) affect afterload independent of resistance [21]; and (3) affect proximal wall shear stress (WSS) and thus potentially have impacts on cellular signaling through mechanotransduction [3]. Recent studies on the arterial compliance show that increased PVR correlates to decreased arterial compliance, which establishes a constant [22, 23] or shorten resistance-compliance time [24–26] during PH progression. The decrease in compliance, such as the proximal stiffening is also important and affects the entire vasculature, as well as increased right ventricular (RV) afterload [27, 28]. These observations indicate that the evaluation of upstream vascular stiffness could play a more prominent role in pulmonary hypertension diagnosis than is currently considered, with the current diagnostic PVR quantifying only the static component of pulmonary hemodynamics. However, the magnitude of these three effects and the overall role of PVS in progressing the disease remain unclear. Although currently there are no treatments aimed towards reducing proximal stiffness, an additional interesting question is raised whether such treatments would be beneficial to improve PH treatment.

Progression of pulmonary hypertension involves continuously changing vascular hemodynamics and remodeling of the proximal and distal arteries. It is understandably difficult to investigate an individual parameter's effect on PH in an *in vivo* study. Here, we propose a modeling procedure to simulate PH progression by dividing it into 4 stages. At each stage we only consider system changes due to varying a single pulmonary vascular parameter, from PVR, elastic modulus (EM), and arterial thickness (*h*), starting with the control condition. We evaluate the role of these three key parameters in modulating input impedance and hemodynamics using a two-dimensional (2D) pulmonary vascular computational model based on *in vivo* and *ex vivo* measurements obtained from normal and chronically hypoxic neonatal calves [1, 29]. We first validate our numerical results on mPAP and the first harmonic of input impedance (*Z*_1_) by comparing animal measurements to 17 calf specific computational models. We then perform predictive studies to assess the effects of PVR, EM, and *h* on the arterial pressure, WSS, impedance and RV power. Finally, the impacts of theoretical and available PH treatments are examined by reversing these parameters, thereby investigating the added effect of targeting proximal vascular remodeling for treatment in PH. We believe this simple but validated model offers important insight of PH progression and might encourage new treatment targets.

## 2. Methods

The ability to develop a unique and validated model for each subject and achieving reasonable solution times that allow for routine simulation are the two critical factors that motivate us to employ numerical techniques, and should enable simpler application of these techniques in both research and clinical environments. Although there have been a variety of numerical models developed by our group and others, ranging in complexity from fully three dimensional, exact geometry models to simple one dimensional bulk flow models, we believe neither fully satisfies these two critical factors. By developing a simple but still subject-specified model, an approach is proposed through which we obtain information needed to study PH progression yet perform validation with a larger number of subjects.

**Figure 1:**
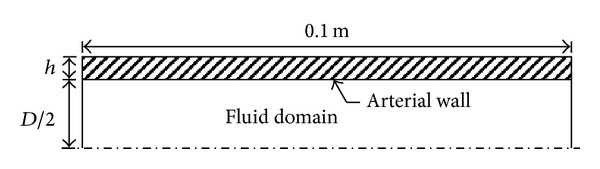
The schematic of the two dimensional symmetric pulmonary artery model (*h* is the thickness of the arterial wall and *D* is the arterial lumen diameter, the length of MPA model is 0.1 m).

Our primary intent here is to examine how hemodynamics and RV power change in PH progression and during treatment. Our model consists of a single elastic tube, representing the larger upstream compliant vessels, which is modeled in two dimensional (2D) axisymmetric coordinates.  Figure 1 shows the schematic of the tube model with diameter *D*, thickness *h*, and a total length of 0.1 m. Three simulation types are performed in this paper: a validation study, a PH progression study, and a PH treatment study. For the first, parameters from seventeen calves (two groups: control and hypertensive) are used to develop subject-specific models and validation occurs through comparison of multiple model results to experimental measurements. The PH progression and treatment studies then utilize mean values from the control and hypertensive groups to assess the impacts of specific parameters on afterload.  Table 1 shows the parameters of the calves in control and hypertensive groups and the mean values employed, as will be explained in more detail below.

## 3. Numerical Model

### 3.1. Fluid Domain

The blood is modeled as an incompressible, laminar, Newtonian fluid with a viscosity of 3.5 mPa·s and a density of 1050 kg/m^3^. At model initiation, there is no flow in the domain as well as no transmural pressure. At the artery model lumen the nonslip condition is applied to the fluid velocity, and normal stress is continuous across the lumen. An axisymmetric boundary is defined at the centerline of the arterial wall, thereby reducing the mesh size, and in turn, the simulation time. The inlet flow rate is transient, with pulse shape obtained from experimental *in vivo* measurements, as explained below. The outlet boundary condition of the flow domain is specified as a resistance boundary condition (32), *R* = *P*/*Q*, with *P* and *Q* being given by as the measurements mPAP and CO, respectively.

### 3.2. Solid Domain

The pulmonary artery wall is considered as an incompressible isotropic linear elastic model with density 1200 kg/m^3^ and Poison's ratio 0.48; the elastic moduli of the models were obtained from *ex vivo* measurements and are discussed further below. The boundary condition at each end is specified as free radial motion and zero longitudinal motion; there is no load on the exterior surface, and only internal coupled fluid pressure/shear stress is applied on the interior surface (fluid structure interface).

### 3.3. Solution Procedure

The CFD-ACE multiphysics package (CFD Research Corporation, CA, USA) and MATLAB (Matworks, Natick, MA) are used to perform the numerical simulations for both fluid and solid domains, including experimental data and results processing. A rectangular mesh is generated for the axisymmetric geometry using CFD-GEOM initially containing uniform fluid and solid elements. The mesh contains 4221 fluid nodes and 1005 solid nodes, with 201 nodes on the fluid-structure boundary. Time integration utilizes a first-order Euler approach with a time step of 2 ms. Details of the solution of the fluid and structural equations can be referred to our previous work [30–32]. Both CFD-VIEW and MATLAB are used to postprocess the resulting simulation results.

Because the resistance boundary condition coupled with wall stiffness allows for fluid storage, the simulation needs to take several cardiac cycles to become periodic. Here, we run each model for 6 continuous cardiac cycles to insure that the simulations are convergent in the transient inlet condition. A serial computer system (AMD Opteron (tm) Processor 280 2.4 GHz, 2.0 G memory) performed the simulations; typical wall time for one model is about 5 hours for a typical simulation time of 3 seconds (6 cardiac cycles).

We carried out mesh validation by comparing our baseline mesh to a 1.5 × refined mesh (9331 fluid nodes, 1806 solid nodes) and verified time convergence by halving the time step (1 ms). A total 6 cardiac cycles are simulated and the fluid pressure/velocity at the end of simulations are compared with and without these temporal and spatial refinements. At nine different locations, defined in the axial direction at the inlet, midpoint, and outlet; along the radial direction at the axis of symmetry, halfway between the axis and the wall; and at the arterial wall, the fluid pressure or velocity has less than 1% change with refined mesh and less than 1% difference with refined time step, respectively.

## 4. Animal Data Acquisition

All studies were performed after the approval by the appropriate Institutional Animal Care and Use Committees. Seventeen newborn male dairy calves (Holstein) weighing between 35 and 50 kg were used. Data obtained from intact calves include the MPA lumen diameter, thickness, and elastic modulus (PVS); the mean MPA pressure (mPAP) and cardiac output (CO), the MPA centerline velocity time history, and pulmonary vascular input impedance (*Z*); several of the experimental measurements were described in previous papers [1, 2]. To make the data acquisition process clearer, these experimental methods are described briefly here.

Our *in vivo* parameters are mPAP, CO, and the MPA velocity time history [2]. A solid-state (Millar SPC-350) catheter placed in each calf MPA via the right jugular using the Seldinger technique was used to obtain pressure waveforms, while a caudal, short-axis view at the 4th intercostal space, 3–6 cm dorsal to the elbow was used to obtain pulse-wave Doppler images of instantaneous blood velocity in the MPA. Cardiac output (CO) was measured with a Swan-Ganz catheter by thermodilution in all animals. PVR was obtained from measured flow rate (CO) and pressures [11]. Finally, pulmonary distal resistance and vascular input impedance (*Z*) were computed from pressure and PW Doppler [11]. We note that although we have only published impedance data derived from echo and pressure measurements taken in the clinic, the post-processing for animal models is identical. *Ex vivo* measurements were taken to obtain the MPA diameter, thickness, and elastic modulus [1]. Extracted calf MPAs were processed into circumferential strips and tested on a standard material testing system (MTS Insight II, Eden Prairie, MN) to obtain *ex vivo* elastic modulus [2] over a wide range of strains. Diameter and thickness were measured using optical methods [4]. Additional work required to implement these data in the model is described below.

## 5. The Use of Experimental Data in the Model

### 5.1. Model Geometry and Elastic Properties

The model geometry and elastic properties, including the diameter, thickness, and elastic modulus, are all obtained from measurements of calf MPAs [1]. For validation, each of the seventeen models used has a subject-specific MPA diameter, thickness and elastic modulus with a single length of 0.1 m. For the simulation studies (progression and treatment), we averaged thickness and elastic modulus values from eight control and nine hypertensive animals to obtain model endpoints. Because there was no statistically significant change in the lumen diameter between control and hypertensive groups, we used one averaged diameter for all simulations. These averaged values are shown in Table 1 and indicate the true physiological changes seen during PH progression.

### 5.2. Inlet Flow Condition

We have previously used our inlet condition in clinical models [28]. Briefly, inlet flow velocity is assumed to be spatially constant, with magnitude determined from calf-specific or generic flow time histories and current inlet cross-sectional area. For the validation models, flow time histories were obtained in a calf-specific manner by combining mean flow measurement (i.e., cardiac output) with MPA midsection blood velocity (pulse-wave Doppler). Velocity histories in five consecutive cardiac cycles were taken, averaged, and scaled to the known mean cardiac output with the area correction factor to obtain a final flow time history [11, 28, 33, 34]. After calf-specific velocity time histories were computed they were used to obtain group-averaged time-histories by first normalizing them in time with respect to their cardiac cycle length. Next, velocity at the normalized time was then averaged on a group-wise basis (8 controls, 9 hypertension). Finally the average cardiac cycle time length was implemented to redimensionalize the time.

**Figure 2:**
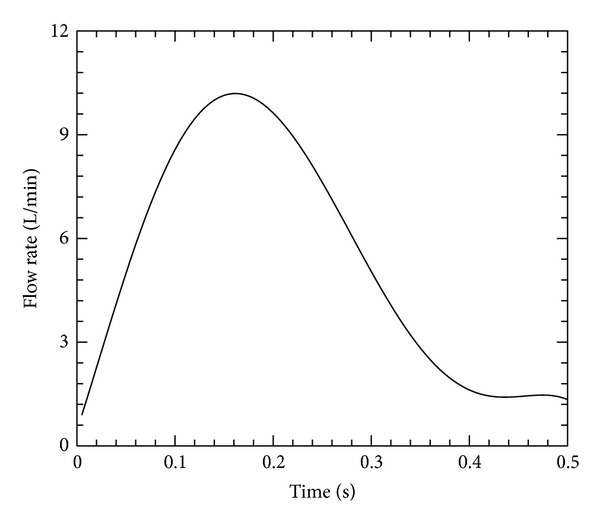
The flow rate applied in the models for control and hypertensive conditions (CO: mean 4.74 L/min, range 0.9–10.2 L/min; HR: 120 Beat/min).

For the predictive studies, we average heart rates of all seventeen calves and use the same heart rate for both control and hypertensive models. Because the heart rate is affected more sensitively by daily activities (such as work, walk, sleep, etc.), its effect on the PH will not be considered in this study [35]. Additionally, there was no statistically significant change in the CO between control and hypertensive groups, and we used one averaged CO and flow waveform for all simulations. The flow waveform used in the predictive models is shown in Figure 2.

### 5.3. Modeling of the Stiffening Process

Based on our recent vascular stiffening research in this animal model [1], there are two stiffening processes involved in PH progression: elastin remodeling and collagen engagement. The elastin remodeling yields an increase of approximately two times the linear elastic Young's modulus of the artery wall at smaller stretch ratios (defined as ratio of instantaneous lumen diameter to initial lumen diameter). Due to the engagement of vascular collagen at higher stretch, the collagen engagement is nonlinear and yields a substantial increase in the incremental material modulus above the engagement stretch (the stretch at which collagen begins to carry load). In our model, each change is simply modeled as a single increase in the linear incremental modulus as measured experimentally with *ex vivo* methods [1].

## 6. Post Numerical Data Processing

MATLAB codes were used (MATLAB V7.7, The Mathworks, Natick, MA) to calculate the WSS, input impedance, and ventricular powers. The WSS is *μ*(∂*u*/∂*r*)_*r*=*R*_ and derived from velocity *u* by fitting the near-wall velocity data with a three-point polynomial function [36]. Here, *μ* is the viscosity of blood, *r* is radial vector, and *R* is the local instantaneous radius of the pulmonary arterial wall. The mean WSS is time-average WSS and the pulse WSS is defined as (WSS_max⁡_ − WSS_min⁡_) in one fully developed cardiac cycle. The wall pressure is obtained at the middle point between inlet and outlet boundaries, the same location of WSS. The definitions of mPAP and PP are similar to those of mean and pulse WSS. Details regarding the calculation of impedance from pressure and flow waveforms may be found in our previous works [11, 28, 33]. RV power (the rate of ventricular work) comprises potential energy and kinetic energy. The mean potential power (mean energy per unit time) and the oscillatory potential power (pulsatile energy per unit time), and the mean kinetic power and oscillatory kinetic power were calculated by using Milnor's methods [20, 35]. The mean power was calculated as *W*_*M*_ = *P*_0_ · *Q*_0_, where *P*_0_ and *Q*_0_ are the zero-frequency components of the pressure and flow moduli, respectively. The oscillatory power was calculated as

(1) $$\begin{array}{c} W_{O}=\frac{1}{2}\sum_{n=1}^{3}{\left|Q_{n}\right|}^{2}\left|Z_{n}\right|\cos\phi_{n}, \end{array}$$

here, *n* represents the harmonic of the fundamental frequency. Finally, total RV power was calculated as

(2) $$\begin{array}{c} W_{T}=W_{M}+W_{O}. \end{array}$$

## 7. Statistics

With the exception of heart rate, all data from experimental measurements are presented as mean ±  SD. For validation, the first harmonic impedance *Z*_1_ and mPAP computed in our seventeen subject-specific models were compared with the results from measurements with Student's *t* test. *P* values refer to the probability associated with the two-tailed Student's *t* test, and results were deemed statistically significant for *P* values less than 0.05. Linear regression and Bland-Altman methods [37, 38] are employed to examine the difference of impedance *Z*_1_ and mPAP between numerical results and measurements and thereby validate the computational modeling approach. Limits of agreement (LoA), defined as the interval expected to contain 95% of the differences, were calculated and are given by the mean difference plus or minus 1.96 standard deviations. 95% confidence intervals were taken around the LoA to evaluate their precision and consistency. MATLAB codes were written (MATLAB V7.7, The Mathworks, Natick, MA) to perform the *t* test, linear regressions, and Bland-Altman analyses.

## 8. Results

### 8.1. Experimental Measurements

Table 1 provides the calf experimental measurements that determined model geometry (arterial lumen diameter and arterial wall thickness), inlet flow rate (heart rate (HR) and cardiac output (CO)), material properties (*ex vivo* elastic modulus), and exit conditions (PVR). The thickness, elastic modulus, PVR, and mPAP are higher in hypertensive than those in control conditions (*P* < 0.05). There was no statistically significant change in the lumen diameter and CO as a result of hypertension. Qualitatively comparing parameters that did change between the control and hypoxic conditions, PVR (208%), elastic modulus (82%) and mPAP (164%) change substantially more than thickness (17%).

### 8.2. Numerical Model Validation

**Figure 3:**
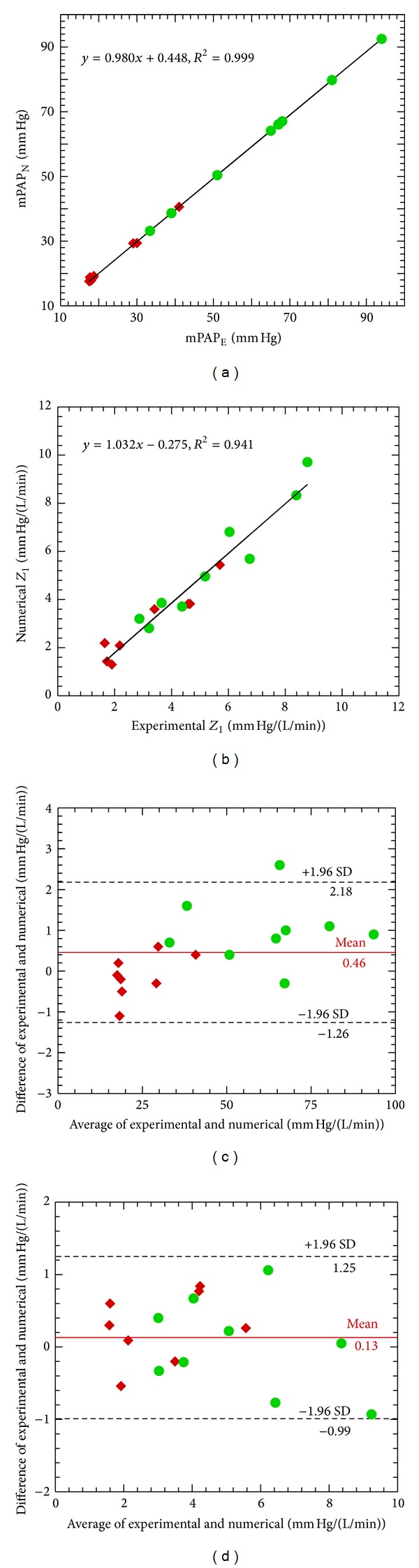
Comparison of the mean main pulmonary arterial pressure (mPAP) and input impedance modulus *Z*_1_ between numerical (N) and experimental (E) results by linear regression (Top) and Bland-Altman (Bottom) methods (◆: Control; *●*: Hypertension).

To validate the artery model, seventeen calf-specific simulations were performed, and the computed first harmonic modulus of input impedance (*Z*_1_) and computed mPAP were compared to experimental measurements.  Figure 3 comprises our validation comparisons. Figures 3(a) and 3(b) display linear regressions between the numerical mPAP_N_ results to experimental mPAP_C_ measurements, and between the numerical and experimental impedance *Z*_1_. The regressions equations are *y* = 0.980*x* + 0.448, *R* ^2^ = 0.999 (*P* < 0.05) for mPAP and *y* = 1.032*x* – 0.275, *R* ^2^ = 0.941 (*P* < 0.05) for the impedance modulus *Z*_1_. Figures 3(c) and 3(d) display Bland-Altman analyses for mPAP and impedance modulus *Z*_1_ between numerical and (gold-standard) experimental results. The Bland-Altman analyses show a bias ± LoA of 0.46 ± 1.74 mm Hg for mPAP and 0.13 ± 1.12 mm Hg/(L/min) for the impedance modulus *Z*_1_. Clearly the bias and LoA for mPAP are small compared to the typical pressure value, while the bias and LoA are more significant for the impedance modulus.

### 8.3. Study of PH Progression

The simulated effect of changes in our key parameters, namely, PVR, EM, and proximal thickness (*h*)—on pulmonary hemodynamics and RV power during PH progression—are studied for both progression (increases in parameters) and treatment (reduction in parameters). First we consider the progression of PH into four stages: (1) control, (2) hyper PVR (PVR: 4.91 → 15.1 mm Hg/(L/min)), (3) hyper EM (EM: 97.1 → 177 kPa), and finally (4) hyper *h* (thickness: 3.38 → 3.95 mm), which are described in Table 2. All other parameters in control and hypertensive conditions are from the averaged experimental measurements in Table 1. We note that due to the preserved flow rates (CO) between the control and hypoxic animal groups, pressure alone acceptably tracts pulmonary hemodynamics in this study.

**Figure 4:**
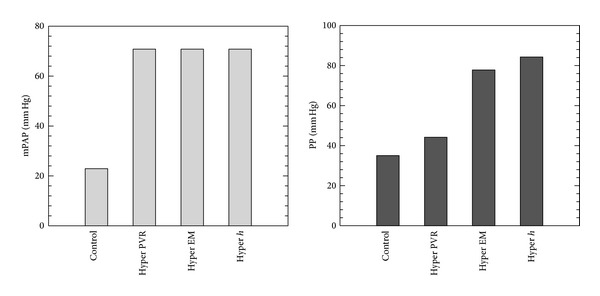
Comparison of proximal pressure for all stages from control to hypertensive conditions during PAH progression (Hyper PVR: PVR from 4.91 to 15.1 mm Hg/(L/min); Hyper EM: EM from 97.1 to 177 kPa; Hyper *h*: arterial wall thickness from 3.38 to 3.95 mm to hypertensive condition).

**Figure 5:**
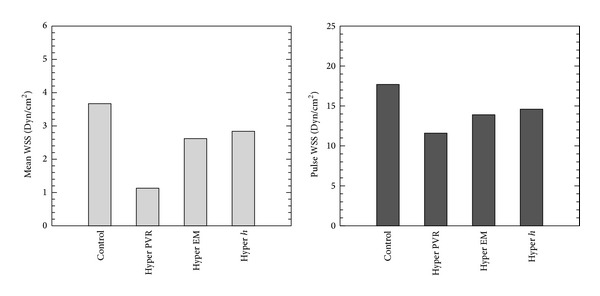
Comparison of WSS for all stages from control to hypertensive conditions during PAH progression (Hyper PVR: PVR from 4.91 to 15.1 mm Hg/(L/min); Hyper EM: EM from 97.1 to 177 kPa; Hyper *h*: arterial wall thickness from 3.38 to 3.95 mm to hypertensive condition).

**Figure 6:**
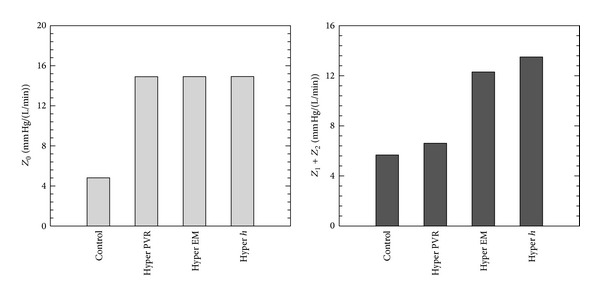
Comparison of input impedance modulus for all stages from control to hypertensive conditions during PAH progression (Hyper PVR: PVR from 4.91 to 15.1 mm Hg/(L/min); Hyper EM: EM from 97.1 to 177 kPa; Hyper *h*: arterial wall thickness from 3.38 to 3.95 mm to hypertensive condition).

Figures 4, 5, and 6 show comparison of proximal pressure, WSS and input impedance for all stages from control to hypertensive conditions during PH progression. For these and all other results, pressures and WSS are obtained at the lumen wall halfway between inlet and outlet boundaries. Increased PVR (208%) yields elevated mPAP and pulse pressure (PP); however, mPAP (209%) increases much more than PP (26.4%). Higher pressure in condition “Hyper PVR” expands the vessel to a larger cross sectional area which in turn lowers the flow velocity. As a result, WSS is reduced both in mean (to 30.9% of control) and pulse components (to 65.4% of control). The increased elastic modulus in condition “Hyper EM” stiffens the arterial wall and alters the pressure pulsatility alone, and at the same time the wall displacement is reduced and WSS is increased. The thicker arterial wall in condition “Hyper *h*” has the same effect as increasing elastic modulus, in that it too increases the pulsatility of pressure and WSS. For the impedance moduli, the *Z*_0_ is more associated with PVR and *Z*_1_, *Z*_2_ are increased as EM and *h* increase. These impedance results agree with previous studies that found that the zero harmonic is associated with the PVR while the higher order harmonics of impedance are affected by PVS.

**Figure 7:**
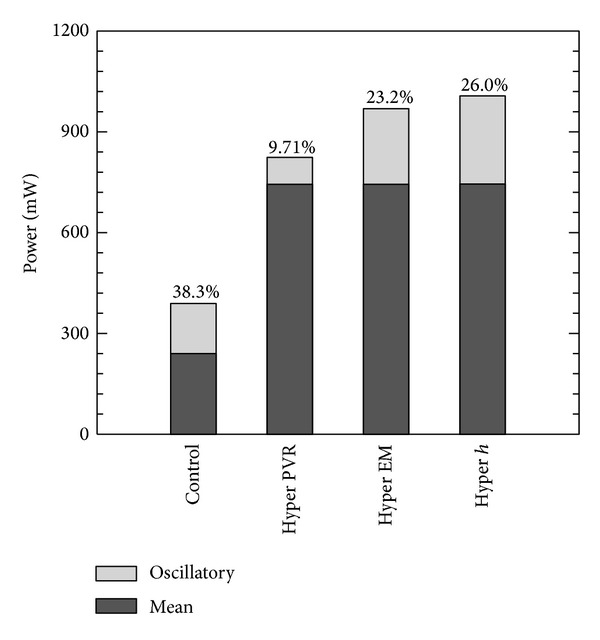
Comparison of RV powers for all stages from control to hypertensive conditions during PAH progression, percentage shown the oscillatory power over total power (Hyper PVR: PVR from 4.91 to 15.1 mm Hg/(L/min); Hyper EM: EM from 97.1 to 177 kPa; Hyper *h*: arterial wall thickness from 3.38 to 3.95 mm to hypertensive condition).

Figure 7 shows comparison of mean/oscillatory and RV power for all stages from control to hypertensive conditions. In the control condition, the oscillatory power contributes 38.3% of the total ventricular power, which agrees with clinical measurements for adults [35] and experimental results from other animal studies [39]. As the PVR increases at the beginning of PH progression, the mean ventricular power increases significantly while the oscillatory power decreases. This unusual relationship between oscillatory power and pulse pressure changes is likely due to the preserved cardiac function applied in the progression model. The increase in EM and *h* contribute mostly to the increase of oscillatory power, while the mean power is kept constant. As the calf progresses to the hypertensive condition, the percentage of oscillatory power over total ventricular power decreases to 26.0%, which again is compatible with previous animal study results [39].

### 8.4. Effect of Treatment on Proximal Vascular Remodeling

Clinical treatment of PH focuses only on decreasing pulmonary vascular resistance, that is, relaxing distal vascular smooth muscle cells. Here, we include this effect and further examine the effects of treatment on individual parameters by applying our key parameter changes to the initially diseased model state. Thus, we start with the final model of our progression simulation, first apply standard clinical treatment—a vasodilator to reduce PVR—and then apply a theoretical treatment that reduces proximal remodeling, thereby lowering proximal elastic modulus and thickness; this sequence is described in Table 3 and results in three treatment models.

**Figure 8:**
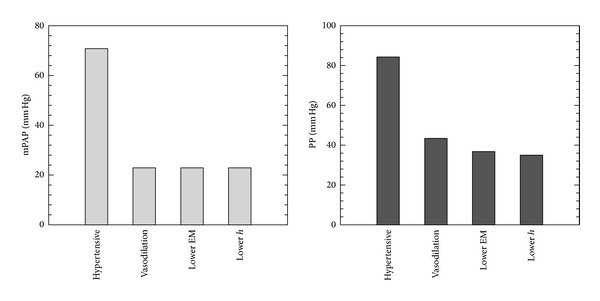
Comparison proximal pressure between vasodilation and proposed PAH treatments on proximal vascular remodeling (Vasodilation: PVR from 15.1 to 4.91 mm Hg/(L/min); Lower EM: EM from 177 to 97.1 kPa; Lower *h*: arterial wall thickness from 3.95 to 3.38 mm to control condition).

**Figure 9:**
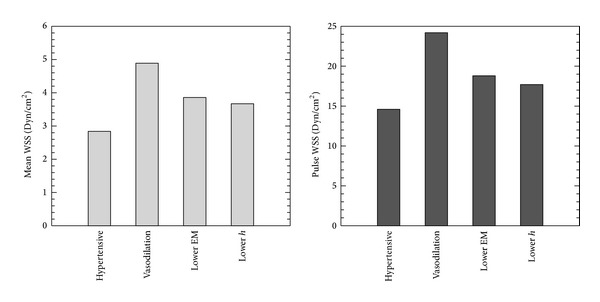
Comparison proximal WSS between vasodilation and proposed PAH treatments on proximal vascular remodeling (Vasodilation: PVR from 15.1 to 4.91 mm Hg/(L/min); Lower EM: EM from 177 to 97.1 kPa; Lower *h*: arterial wall thickness from 3.95 to 3.38 mm to control condition).

**Figure 10:**
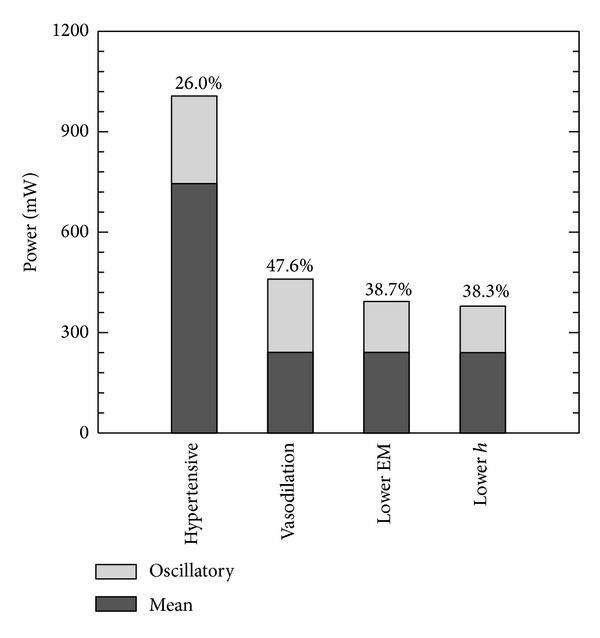
Comparison RV power between vasodilation and proposed PAH treatments on proximal vascular remodeling, percentage shown the oscillatory power over total power (Vasodilation: PVR from 15.1 to 4.91 mm Hg/(L/min); Lower EM: EM from 177 to 97.1 kPa; Lower *h*: arterial wall thickness from 3.95 to 3.38 mm to control condition).

Figures 8 and 9 show the comparison of pressure and WSS for these individually changing parameters in PH treatment. The vasodilator treatment, which reduces PVR, is also predicted to decrease both mPAP and pulse pressure dramatically. The treatments to address proximal vascular remodeling (thereby reducing the elastic modulus and thickness) do not alter mPAP but do further decrease the pulse pressure. The decreased PVR in PH treatment actually increases mean and pulsatilite WSS, while treatment on proximal stiffness could reduce these changes.  Figure 10 shows comparison RV power between vasodilation and theoretical proximal remodeling treatments; PVR changes significantly mean power, which contributes most to total ventricular power. Although the oscillatory component makes only a small contribution to the total, decreasing elastic modulus and arterial thickness does further reduce oscillatory power, thereby modestly reduce RV afterload. These observations suggest that decreasing or reversing proximal vascular remodeling could benefit PH treatment, in addition to the standard vasodilator approach.

## 9. Discussion

Current clinical evaluation and treatment of PH focuses merely on the resistive portion of the pulmonary circuit, but many clinical studies are demonstrating that proximal stiffness strongly affects disease progression. Here, we develop and validate a simple 2D numerical model to explore the impacts of PVR and PVS in the progression and treatment of PH. The validated model results for WSS, RV power, and impedance have implications for further basic science and clinical study, as described below.

To validate the model, we compared mPAP and input impedance modulus *Z*_1_ between the numerical simulation and experimental measurements. Both regressions show excellent goodness of fit, which alone does not indicate correspondence; however, combined with the Bland-Altman results these comparisons support the idea that the model reasonably represents global hemodynamics through the use of calf-specific model parameters. Further, general agreement exists between our model and other experimental or clinical WSS and proximal deformation results [3, 4, 40]. Finally, RV powers [35, 39] and input impedance both displayed trends which are seen in other animal models [33], as described in the results section. Such correspondence, in prediction of both global hemodynamics and general trends in local features such as WSS, gives us greater confidence in our simulated results.

Our simulation results provide evidence that the different components of afterload play unique roles in progressing PH. Increasing PVR yields severe mPAP and modest pulse pressure increases, while increasing stiffness results in only increases to pressure pulsatility. Because WSS is associated with cross sectional area, it tends to decrease during PH progression, and is associated with a more greatly distended vessel. Such changes in WSS suggest that mechanotransduction, which correlates the cellular, molecular, and tissue behaviors and pulmonary hemodynamics in blood vessels, could potentially play a role in disease progression [41]. Of note is the observation that the two components of afterload have opposite effects on WSS.

Regarding RV power, clearly, changes in PVR and elastic modulus play more important roles than changes in thickness. Previous clinical and experimental studies [35, 39] show that the oscillatory power is less of the total RV power when the PH worsens, in agreement with our progression simulations. During vasodilator treatment, we see that the oscillatory power contributes more substantially to total RV power (47.6% versus 26% untreated); thus, for decreasing the ventricular power, the reduction of proximal vascular remodeling could be a future diagnostic target. However, even as the treatment simulation progresses to incorporate our theoretical remodeling reduction, only modest further reductions are obtained in RV power. Given the strong association between stiffness and clinical outcomes, this suggests that increased stiffness may have other deranging effects on the pulmonary circulation, such as decoupling the RV from the vessels [42].

Finally, the input impedance, which is derived from the pressure and flow profiles in pulmonary circulation, is shown as an accurate single measurement of global pulmonary hemodynamics. Several recent studies have shown that impedance can best quantify overall hemodynamics, in that it quantifies both major components, PVR and PVS. If common clinical or experimental parameters can be obtained on a noninvasive basis [33, 43, 44], our model might be used to compute impedance, and thereby provide further insight into the level and degree of PH progression through simulating patient power, general shear conditions, and exploring the degree of proximal vascular remodeling.

Overall, the effects of PVS, PVR, and proximal artery geometries on the pulmonary hemodynamics and RV power, as well as RV afterload are studied during PH progression and proposed treatment. PVS and PVR have more influences on the pulmonary hemodynamics and TV power. However, the increased PVR contributes to the increase of RV afterload, which only reflects the steady component of total RV power. The proximal stiffness is an important determinant of the oscillatory components of pulmonary hemodynamics and RV power. This agrees to the previous finding that an increase in proximal stiffness is an excellent predictor of mortality in patients with PAH and right heart failure [16, 17].

## 10. Limitations

With accurate experimental parameters, this simple 2D axisymmetric numerical model represents a reasonable advance in calf-specific models that are applied to larger numbers of subjects. However, as previously discussed [28], several limitations remain. 3D effects, such as nonsymmetrical spanwise flow, and complex geometry are ignored in this axisymmetric model and would influence flow pattern in the arteries and in turn affect WSS. However, our previous modeling results suggest such spanwise flows are small compared to the streamwise velocities. Although dynamic behavior of the arterial wall might be considered as linear (i.e., using an incremental modulus approach, as we have done here) during *in vivo* pulsatile flow transportation, our model could further include more realistic viscoplastic wall behavior, especially in the severe PH condition where collagen engagement plays a more important role on vascular behavior. This limitation affects the accuracy of our model, such as in the control condition, the oscillatory power contributes 38.3% of the total ventricular power, which agrees with clinical measurements for adults [35] and experimental results from other animal studies [39] but higher than the results shown in a recent study [27]. Further, our resistance boundary condition simplifies the complex flow impedance presented by the distal vasculature to merely a resistance condition; however, the distal circulation is believed to contribute less compliance to the entire circuit, and despite using a resistance exit condition, model compliance allows for the existence of diastolic flow. Finally, our other assumptions, including constant heart rate, CO, and blood viscosity, primarily enable us to better focus our model on investigating the effect of PVR and PVS in progressing PH; such simplifications appear reasonable for these single branch models given lack of measured significant differences in the animal model. Based on all these simplifications, we have developed a numerical model that may be more easily implemented clinically with reduced construction time and runtime.

## 11. Conclusion

In this paper, the effects of PVR and PVS (elastic modulus and thickness) on pulmonary hemodynamics in PH progression and treatment were examined in a 2D numerical model. The model was first validated using specified data from calves in control and hypertensive conditions. In simulated disease progression, the model suggests that PVR, elastic modulus, and thickness have quantitatively different effects on pulmonary pressures, RV afterload, and power. The combined changes are shown to increase the mean and pulsatile component of hemodynamics while decreasing WSS, which supports the idea of WSS derangement in the PH condition. In simulated disease treatment, the model suggests that targeting proximal vascular remodeling, and in turn stiffness, may aid in recovery by further reducing RV afterload and power requirements. Finally, the relatively small impacts that stiffness has on RV parameters suggest that, given its newly found prominence in clinical outcomes prediction, it may affect RV function through yet undetermined pathways.

## Figures and Tables

**Table 1:** Parameters from experimental measurements to develop and validate numerical models, 8 in control and 9 in hypertensive condition. Mean ± SD performed for each condition (arterial wall thickness, elastic modulus, PVR, and mPAP) and all cases (diameter, CO, and HR).

	No.	Thickness (mm)	Elastic modulus (kPa)	PVR (mm Hg/(L/min))	mPAP (mm Hg)	Diameter (mm)	CO (L/min)	HR (Beat/min)
Control	165	3.68	72.7	4.91	18.5	16.0	3.88	114
	166	3.44	136	4.79	29.0	19.7	6.06	147
	174	3.04	138	8.88	41.0	18.2	4.62	130
	175	2.94	105	5.17	30.0	21.8	5.80	113
	176	3.34	98.4	3.83	17.5	19.8	4.70	100
	177	3.71	64.1	5.01	18.7	19.7	3.80	94
	180	3.34	77.2	3.64	17.7	19.9	5.22	114
	181	3.52	86.0	3.08	18.0	18.9	5.85	113

Mean ± SD		3.38 ± 0.28	97.1 ± 32.6	4.91 ± 1.77	23.8 ± 8.7			

Hyper	167	4.19	185	14.3	68.0	24	3.45	125
	168	3.50	113	19.7	39.0	18.75	5.21	118
	169	5.12	92.6	7.48	67.0	20.4	5.12	135
	170	3.60	116	13.1	51.0	20.46	4.12	122
	171	3.98	252	12.4	65.0	22.55	3.87	131
	172	3.84	267	16.8	94.0	19.94	5.36	148
	173	3.42	187	17.5	67.0	18.8	3.52	139
	178	3.90	245	19.0	81.0	20.65	5.20	117
	179	3.97	136	15.6	33.4	22.22	4.83	132

Mean ± SD		3.95 ± 0.51	177 ± 66	15.1 ± 3.8	62.8 ± 19.2	20.1 ± 1.9*	4.74 ± 0.83*	120 ± 15*

*The averaged value for all control and hypertensive cases.

**Table 2:** Four stages from control to hypertensive conditions during PAH progression (each stage one parameter increased from previous stage).

Control	All parameters from control state (Table 1)
Hyper PVR	PVR: 4.91 → 15.1 mm Hg/(L/min)
Hyper EM	EM: 97.1 → 177 kPa
Hyper *h *	*h*: 3.38 → 3.95 mm

**Table 3:** Proposed treatments on PAH with vasodilation and proximal vascular remodeling (each proposed treatment targeted to reduce one parameter from previous stage).

Hypertensive	All parameters from hypertensive state (Table 1)
Vasodilation	PVR: 15.1 → 4.91 mm Hg/(L/min)
Lower EM	EM: 177 → 97.1 kPa
Lower *h *	*h*: 3.95 → 3.38 mm
